# Hypertensive Emergency During Dialysis: A Paradoxical Physiologic Response

**DOI:** 10.7759/cureus.60304

**Published:** 2024-05-14

**Authors:** Steven Imburgio, Anne Arcidiacono, Lauren Klei, Kylie Oppegaard, Anmol S Johal, Ndausung Udongwo, Palak Patel, Mayurkumar Patel

**Affiliations:** 1 Internal Medicine, Jersey Shore University Medical Center, Neptune City, USA; 2 Cardiology, Morehouse School of Medicine, Atlanta, USA; 3 Cardiology, Jersey Shore University Medical Center, Neptune City, USA; 4 Nephrology, Jersey Shore University Medical Center, Neptune City, USA

**Keywords:** case report, intradialytic hypertension, intradialytic hypotension, hemodialysis, hypertensive emergency

## Abstract

Most end-stage renal disease patients experience a reduction in blood pressure during their hemodialysis session compared to predialysis. Surprisingly, a small subset of patients will experience an unusual physiological response to dialysis that results in a paradoxical increase in blood pressure. We discuss a case that involved an exaggerated elevation in blood pressure, ultimately requiring immediate cessation of dialysis and admission to the intensive care unit for intravenous treatment of a hypertensive emergency. This case serves as a framework to introduce the infrequently discussed concept of intradialytic hypertension. The underlying pathogenesis is poorly understood with multiple theoretical etiologies including activation of the renin-angiotensin-aldosterone system, imbalances in circulating levels of endothelium-derived mediators, clearance of antihypertensive medications, increased cardiac output, and changes in arterial thickness. It is important to be cognizant of this phenomenon as emerging evidence suggests that patients with any elevation in blood pressure during hemodialysis have increased rates of both short-term and long-term mortality.

## Introduction

A hypertensive crisis is a common patient presentation in the emergency department (ED) and is characterized by a drastic acute increase in blood pressure above 180/120 mmHg [1]. The two major subtypes include hypertensive urgency and hypertensive emergency [2]. Hypertensive urgency is classified as a rapid rise in blood pressure with no associated signs of end-organ damage [3]. These patients have not been shown to benefit from aggressive therapy in the hospital setting and can often be treated as an outpatient with oral antihypertensives [4]. A hypertensive emergency, on the other hand, is a potentially life-threatening form of severely elevated blood pressure with the involvement of cardiac, renal, neurological, or other organ injuries [5]. Prompt recognition and treatment with intravenous antihypertensives are paramount to safely lowering blood pressure and reducing patient morbidity and mortality [6]. The rapid onset of a hypertensive crisis often points to an inciting event superimposed on poorly controlled hypertension [7]. Common triggers include antihypertensive noncompliance, recreational drug use, and medical conditions that produce an extremely sympathetic response [8]. Identifying and correcting these underlying triggers is critical to preventing future episodes [9]. We present a rare case of hypertensive emergency paradoxically brought on by a session of hemodialysis which required admission to the intensive care unit and immediate intravenous treatment. Additionally, we explore the possible mechanisms that can explain this unexpected physiologic response.

## Case presentation

A 77-year-old female with a past medical history significant for end-stage renal disease (ESRD) secondary to hypertensive arteriolar nephrosclerosis on a three-times-weekly hemodialysis schedule for the past five years and chronic uncontrolled hypertension on valsartan 320 mg daily and labetalol 200 mg twice daily presented from a dialysis center due to elevated blood pressure. She arrived at the dialysis center on a Monday as part of her first of three times weekly schedule. Her blood pressure prior to starting hemodialysis was recorded as 116/58 mmHg after taking her valsartan and labetalol as prescribed, and her average fluid gain was approximately two liters. The patient reported that within minutes of starting her scheduled hemodialysis, staff at the facility notified her that her systolic blood pressure had risen over 220 mmHg. She experienced acute onset of nausea, vomiting, and an occipital headache. The hemodialysis treatment was stopped and she was transferred to the hospital directly from the dialysis facility for the management of her symptomatic hypertension. After interviewing the patient, she reported taking her antihypertensives as prescribed and receiving her morning doses prior to hemodialysis. In the ED, blood pressure was noted to be 233/109 mmHg with all other vitals within normal limits. Multiple repeat blood pressure measurements showed sustained systolic blood pressure readings over 230s. On exam, the patient was ill-appearing, pale, and actively experiencing multiple episodes of non-bloody, nonbilious emesis. The patient was alert and oriented with no neurological focal deficits. A cardiopulmonary exam revealed regular heart rate and rhythm, no murmurs, gallops, or adventitious lung sounds were appreciated. Labs demonstrated a creatinine of 3.50 mg/dL (0.55-1.02 mg/dL), calcium of 8.2 mg/dL (8.7-10.4 mg/dL), and an eGFR of 13 mL/min/1.73m*2 (>60 mL/min/1.73m*2) (Table 1).

**Table 1:** Laboratory investigations on presentation eGFR: estimated glomerular filtration rate

Blood chemistry	Value	Reference range
White blood cells	9.4	4.5-11.0 * 10^3^/µL
Red blood cells	4.50	4.50-5.30 mcL
Hemoglobin	10	12.0-17.5 g/dL
Hematocrit	25.4	36.0-53.0%
Platelet count	262	140-450 * 10^3^/µL
Creatinine	3.50	0.55-1.02 mg/dL
eGFR	13	>60 mL/min/1.73m^2^
Calcium	8.2	8.7-10.4 mg/dL
Phosphorus	4.5	2.8-4.5 mg/dL
Aspartate aminotransferase	32	8-33 U/L
Alanine aminotransferase	51	7-56 U/L
Alkaline phosphatase	101	44-147 IU/L

A non-contrast computed tomography scan of the head revealed no acute intracranial process. Urine drug screen did not demonstrate any illicit drug use. The patient’s blood pressure was unresponsive to intravenous hydralazine and labetalol pushes in the ED. She was subsequently started on a clevidipine drip and transferred to the medical intensive care unit for hypertensive emergency induced by hemodialysis. The patient’s home oral antihypertensives were restarted and she was weaned off the clevidipine drip the following day. She was safely downgraded to the medical wards after observing a trend of adequately controlled blood pressure recordings on multiple readings.

A work-up for secondary causes of the hypertensive crisis was negative including renal artery ultrasound with Doppler, aldosterone-renin ratio, and urine total metanephrines. Nephrology was consulted for inpatient hemodialysis management. No intradialytic hypertension was reported at the following inpatient hemodialysis session. The patient had an uncomplicated hospital course and was safely discharged with outpatient follow-up with cardiology and nephrology.

## Discussion

In the majority of ESRD patients, hemodialysis results in a decrease in blood pressure [10]. Typically, blood pressure will drop by 28-40 mmHg on average, with the sharpest decline occurring during the first quarter of hemodialysis [11]. This rapid drop in blood pressure can be so drastic that patients will report symptoms of hypotension in approximately 20% to 30% of all hemodialysis sessions [12]. Most of these episodes are reversible by decreasing the filtration rate, slowing the rate of blood flow, placing the patient in the Trendelenberg position, or returning the intravascular volume [12]. Intradialytic hypotension is a physiologic response that occurs when the rate of volume removal during ultrafiltration exceeds the patient’s inherent rate of plasma refilling [13,14]. Decreased intravascular blood volume results in less preload and reduced cardiac filling [15]. Typically, blood pressure is maintained through mechanisms that include increased plasma refill, vascular resistance, and cardiac output [11,16]. The pathophysiology of hypotension in many dialysis patients involves inadequate cardiovascular compensatory mechanisms [17]. For example, individuals with underlying cardiac disease may be unable to adequately increase myocardial contractility or heart rate during dialysis sessions [18].

In a small subset of patients, a paradoxical increase in blood pressure can be observed during or shortly after hemodialysis [19]. Current evidence suggests that intradialytic hypertension occurs in approximately 5-15% of hemodialysis treatments [20]. Historically, this phenomenon has been viewed as a benign side effect. However, recent evidence suggests that patients with elevated blood pressure readings from their pre-hemodialysis baseline experience worse clinical outcomes [21]. In the short term, hemodialysis patients with intradialytic hypertension had increased rates of 30-day morbidity and mortality [22]. One retrospective cohort study of 113,255 ESRD patients by Park et al. found that any rise in blood pressure during hemodialysis was also associated with significantly increased all-cause mortality at five years [23]. Another study by Singh et al. demonstrated similar findings with a higher adjusted risk of death reported in individuals who experienced any systolic blood pressure increase from pre-hemodialysis to post-hemodialysis [24].

While the pathophysiology of intradialytic hypertension is not clearly understood, it is suspected that excessive volume removal triggers this hemodynamic response [19]. Inadequate hemodynamic compensatory mechanisms such as shunting to the central circulation, enhanced vascular resistance in the splanchnic region, increased arterial tone, and increased cardiac output can result in increased blood pressure [25]. Sympathetic overactivity, the renin-angiotensin-aldosterone system (RAAS), endothelium-derived mediators, erythropoietin-stimulating agents, dialysis-specific factors, and arterial stiffness have also been hypothesized to play a role in intradialytic hypertension [26]. In terms of sympathetic overactivity, it was shown that patients with chronic kidney disease on hemodialysis have increased sympathetic nerve activity, which could potentially lead to increased blood pressure [25,27]. Volume reduction during hemodialysis sessions can cause increased activation of RAAS and a subsequent increase in blood pressure [28]. El-Shafey et al. conducted a study showing that imbalances in endothelin-1 and nitric oxide levels could also cause rebound hypertension when endothelin-1 levels increase or nitric oxide levels decrease [29]. The use of erythropoietin-stimulating agents during sessions can lead to an increase in endothelin-1 and an associated increase in blood pressure [30]. Clearance of anti-hypertensive medications during dialysis may also play a significant role in intradialytic hypertension [31]. Another possible mechanism involves the high levels of sodium and calcium in dialysate, which can affect cardiac contractility and output, along with a decrease in arterial compliance [32,33]. Arterial stiffness from underlying arteriosclerosis can contribute to hypertension during dialysis, as shown in a cohort study by Mourad et al., which showed that patients with an increase in mean arterial pressure during hemodialysis had higher pulse wave velocity, indicating increased arteriosclerosis [34]. While there are many possible mechanisms to explain the pathogenesis of intradialytic hypertension, additional studies are necessary to assess the predominant underlying etiology.

## Conclusions

Many ESRD patients experience decreased blood pressure during hemodialysis due to cardiovascular physiologic mechanisms that are unable to adequately compensate for the reduced intravascular blood volume. Rarely, hypertension can present as a paradoxical response to hemodialysis. The pathogenesis of intradialytic hypertension is complex and an area of ongoing research. Current evidence suggests that this phenomenon carries clinical significance, with worse short-term and long-term mortality for this patient demographic. Better identification of individuals who experience intradialytic hypertension is needed to improve patient outcomes, along with further exploration regarding possible interventions and management.
